# *Providencia entomophila* sp. nov., a new bacterial species associated with major olive pests in Tunisia

**DOI:** 10.1371/journal.pone.0223943

**Published:** 2019-10-22

**Authors:** Ines Ksentini, Houda Gharsallah, Maryam Sahnoun, Christina Schuster, Sirine Hamli Amri, Rim Gargouri, Mohamed Ali Triki, Mohieddine Ksantini, Andreas Leclerque

**Affiliations:** 1 Tunisian Olive Institute, University of Sfax, Sfax, Tunisia; 2 Institute for Microbiology and Biochemistry, Hochschule Geisenheim University, Geisenheim, Germany; 3 Institute for Sustainable Plant Protection (IPSP), National Research Council (CNR), Portici, Italy; University of Minnesota, UNITED STATES

## Abstract

Bioprospection for potential microbial biocontrol agents associated with three major insect pests of economic relevance for olive cultivation in the Mediterranean area, namely the olive fly, *Bactrocera oleae*, the olive moth, *Prays oleae*, and the olive psyllid, *Euphyllura olivina*, led to the isolation of several strains of readily cultivable Gram-negative, rod-shaped bacteria from Tunisian olive orchards. Determination of 16S ribosomal RNA encoding sequences identified the bacteria as members of the taxonomic genus *Providencia* (*Enterobacterales; Morganellaceae*). A more detailed molecular taxonomic analysis based on a previously established set of protein-encoding marker genes together with DNA-DNA hybridization and metabolic profiling studies led to the conclusion that the new isolates should be organized in a new species within this genus. With reference to their original insect association, the designation “*Providencia entomophila*” is proposed here for this hypothetical new taxon.

## Introduction

The cultivation of olives and the production of olive oil are of outstanding economic importance for several Mediterranean countries including Tunisia. Main pest insects threatening olive cultivation in these countries are the olive fly, *Bactrocera oleae* (Rossi) (Diptera; Tephritidae), the olive moth, *Prays oleae* (Bernard) (Lepidoptera; Praydidae), and the olive psyllid, *Euphyllura olivina* (Costa) (Hemiptera; Psyllidae). The latter pest is particularly serious around costal areas, where incomplete stages attack flower clusters in high abundance as, e.g., 3 to 6 larvae per bunch and have been reported to cause the loss of 25.9% of the harvested fruits [1].

Due to the enrichment of lipophilic compounds in olives and olive oil, the application of chemical insecticides is particularly problematic in the protection of olive orchards from pest insects [2, 3]. A possible alternative could be the development of microbial control agents or methods based on the action of, e.g., entomopathogenic bacteria. Within the framework of a previous bioprospection effort for bacteria associated with the olive fly, the olive moth or the olive psyllid in Tunisian olive orchards, several bacterial isolates had on the basis of 16S rRNA gene partial sequence comparisons been provisionally assigned to the taxonomic genus *Providencia* [4].

Gram-negative rod-shaped *Providencia* bacteria (*Enterobacterales; Morganellaceae*) [5] have been most notably perceived as natural components of the human gut microflora. The taxonomic genus *Providencia* is currently sub-divided into nine recognized species, namely *P*. *alcalifaciens*, *P*. *burhodogranariea*, *P*. *heimbachae*, *P*. *rettgeri*, *P*. *rustigianii*, *P*. *sneebia*, *P*. *stuartii*, *P*. *thailandensis*, and *P*. *vermicola* [6–12]. Moreover, the new species *P*. *huaxiensis* has been proposed recently [13]. Several of these species as, e.g., *P*. *rettgeri*, *P*. *alcalifaciens* or *P*. *stuartii* are of medical importance as opportunistic pathogens causing gastric disturbations (“traveller’s diarrhoea”) or urinary tract infections [9, 14]. Moreover, *Providencia* bacteria have previously been found associated with and pathogenic to several species of tephritid and drosophilid flies [11, 15–17], but not the olive fly.

In molecular taxonomy studies of *Providencia* bacteria, a Multilocus Sequence Analysis (MLSA) scheme made up of five protein-encoding marker genes (*fusA*, *gyrB*, *ileS*, *lepA*, and *leuS)* has been employed previously in addition to 16S ribosomal RNA encoding sequences [11]. On the basis of 16S rRNA gene and MLSA data together with DNA-DNA hybridization and metabolic profiling studies, the present investigation claims the introduction of a novel species of *Providencia* bacteria.

## Materials and methods

### Bacterial isolation and cultivation

Major olive tree insect pests (namely the olive fly *Bactrocera oleae*, the olive moth *Prays oleae*, and the olive psyllid *Euphyllura olivina*), olive leaves and fallen and suspended *B*. *oleae* or *P*. *oleae* infested olive fruits were collected from Tunisian olive plantations, more exactly from three locations around Sfax (Amra, Ouled-Msallem, and Kerkennah) and one location near Sidi-Bouzid (Table 1). All samplings were made in private olive orchards, with full permission from the respective owners. Samples were surface sterilized with 70% ethanol for 30 sec, rinsed two times with sterile distilled water for 30 sec, and stored at 4°C prior to processing. Bacteria were isolated using the dilution plate method. Briefly, each sample was ground using a micro-pestle in sterilized distilled water. Sample supernatants were serially diluted 10-fold and dilutions were spread on Luria Bertani medium (LB: 10 g/l tryptone, 5 g/l yeast extract, 10 g/l sodium chloride, pH = 7.0). Plates were incubated for 24h to 48h at 32°C and bacterial colonies on the medium were individually identified by appearance, color and–after pure culture isolation—smell. Cultures were purified by serial sub-cultivation and preserved on LB medium for morphological and molecular identification and biochemical characterization. After resistance of the isolates to tetracycline had been noted, isolates were further sub-cultivated on LB medium containing 50 μg/ml of this antibiotic.

**Table 1:** Bacterial isolates investigated in this study.

Isolate designation	Material of origin	Geographic origin GPS coordinates (latitude / longitude)
**IO-6**	*Prays oleae* larva	Ouled Msallem, Jebeniana, Sfax, Tunisia; 34.972805 / 10.792768
**IO-19**	*Prays oleae* leaf gallery	Ouled Msallem, Jebeniana, Sfax, Tunisia; 34.972805 / 10.792768
**IO-20**	*Bactrocera oleae* adult	El Amra, Sfax, Tunisia; 34.973160 / 10.896932
**IO-23**	*Bactrocera oleae* adult	El Amra, Sfax, Tunisia; 34.973160 / 10.896932
**IO-24**	*Euphyllura olivina* larva	Ouled Msallem, Jebeniana, Sfax, Tunisia; 34.972805 / 10.792768
**IO-27**	Olive fruit damaged by *Bactrocera oleae*	Sidi Bouzid, Tunisia; 35.021314 / 9.443128
**IO-28**	Olive fruit damaged by *Bactrocera oleae*	Sidi Bouzid, Tunisia; 35.021314 / 9.443128
**IO-38**	*Euphyllura olivina* larva	Kerkennah, Sfax, Tunisia; 34.649902 / 11.017734

### Growth characteristics and antibiotic susceptibilities

Isolated bacteria were grown at 28°C on LB agar, on LB agar containing X-Gal, and on MacConkey agar. Antibiotic susceptibilities of isolate IO-23 were tested at 28°C on LB agar containing 50ug/ml ampicillin, 50ug/ml kanamycin, 100ug/ml streptomycin or tetracycline at concentrations ranging from 10ug/ml to 200ug/ml. Moreover, non-selective LB plates carrying isolate IO-23 were incubated at 23°C, 28°C, 32°C, 37°C, and 42°C.

### DNA extraction and marker amplification

Bacterial DNA was extracted using the DNeasy Blood and Tissue kit (Qiagen) according to the standard protocol provided by the manufacturer for DNA extraction from Gram-negative bacteria. DNA sample quality was evaluated using a NanoDrop NT-1000 UV spectrophotometer. Almost complete 16S ribosomal RNA genes and internal partial sequences of five MLSA markers encoding translation elongation factor EF-G (*fusA)*, DNA gyrase subunit B *(gyrB)*, isoleucyl-tRNA synthetase *(ileS)*, translation elongation factor EF-4 (*lepA)*, and leucyl-tRNA synthetase *(leuS)* were amplified with an Eppendorf “Mastercycler ep-gradient” using Taq DNA polymerase (New England Biolabs). PCR product sizes were controlled by agarose gel electrophoresis, and PCR products were purified using the Qiaquick PCR purification kit (Qiagen). Sanger sequencing of purified PCR products was performed by Starseq GmbH (Mainz, Germany) using PCR and additional sequencing primers (S1 Table).

### Phylogenetic reconstruction

Raw sequence data were combined into a single consensus sequence for each bacterial isolate and marker using version 6 of the MEGA program [18]. For each marker, the consensus sequence obtained for isolate IO-19 was used as query in BlastN searches [19–20] for similar GenBank database entries across both the completely annotated and draft genome sequences associated with the genus *Providencia* (i.e. GenBank taxid 586) as well as by free searches across single sequence entries. For the latter, identified orthologous GenBank entries were sorted by decreasing sequence similarity percentages and all entries covering at least 80% of the query sequence that were assigned i) to a determined *Providencia* species for the 16S ribosomal RNA gene and ii) to the genus *Providencia* for the protein-encoding MLSA markers were retained for phylogenetic reconstruction. For simultaneous comparison of the five MLSA markers by means of a concatenated sequence, reference strains were considered if sequence data were available for at least four marker genes.

DNA sequences were aligned using the CLUSTAL W function [21] as implemented in the MEGA 6 software package. The Tree-Puzzle 5.2 software [22] was used to estimate data set specific parameters as nucleotide frequencies, the percentage of invariable sites, the transition/transversion ratio and the alpha-parameter for the gamma-distribution-based correction of rate heterogeneity among sites. Pairwise sequence similarity percentages were assessed from a p-distance matrix calculated in MEGA 6 from unfiltered nucleotide sequence data under pairwise deletion of alignment gaps and missing data.

Phylogenies were reconstructed using two algorithms: i) by the maximum likelihood (ML) method as implemented in the PhyML software tool [23] using the Hasegawa–Kishino–Yano model of nucleotide substitution [24] under the assumption of a gamma-distribution-based model of rate heterogeneity [25] allowing for eight rate categories, and ii) by a p-distance matrix-based neighbor joining (NJ) method as implemented in MEGA 6. For both reconstruction methods, tree topology confidence limits were explored in non-parametric bootstrap analyses over 1,000 pseudo-replicates.

### DNA-DNA hybridization

Bacterial DNA isolation and DNA–DNA hybridization was performed by the German Collection of Microorganisms and Cell Cultures (DSMZ) according to [26–28] using a model Cary 100 Bio UV/VIS-spectrophotometer.

### Metabolic profiling

Metabolic characteristics of bacterial isolates were determined using API 20E test strips (bioMérieux). For inoculation, freshly grown single bacterial colonies were resuspended in sterile distilled water and added to the strips according to the standard protocol provided by the manufacturer. Assay results were documented after 24h and 48h incubation at 36°C. The test was performed twice for isolate IO-23 and once for further Tunisian isolates and internal control strains, i.e. *P*. *heimbachae* DSM 3591T, *P*. *vermicola* DSM 17385T, *P*. *sneebia* DSM 19967T, and *P*. *burhodogranariea* DSM 19668T.

### Bioassays against the olive fly, *Bactrocera oleae*

For bioassays, bacterial isolates were grown in liquid LB medium for 24 h or 48 h. Grown cultures were separated by centrifugation into cells and cell-free culture supernatant prior to virulence assays.

In order to collect olive fly pupae, infested olive fruit were placed on top of sieved sand filled into plastic boxes that were held under controlled conditions (25 ± 1°C; 60 ± 10% R.H.; Light/Darkness: 16 h /8 h). Boxes were controlled daily in order to collect new pupae that were transferred to new muslin-covered plastic boxes held under the same conditions and checked daily until flies emerged. Newly emerging adults were sampled in groups of five (n = 5), fed with honey and used for bioassay within 24 h. Flies were sprayed either with 1ml of bacterial culture supernatant or with 500 μg of bacterial cells resuspended in 5ml of sterile LB medium. Three replicates were performed for each treatment, and the control was performed by spraying of sterile LB medium. Flies were checked daily for their mortality up to the 7^th^ day post-inoculation.

## Results

### Growth characteristics and antibiotic susceptibilities

When grown at 28°C on LB agar, on LB agar containing X-Gal, and on MacConkey agar, isolated bacteria were cream coloured, opaque, glossy, smooth, and convex in appearance. As assessed after 48 h incubation on LB agar, isolate IO-23 grew faster at 32°C and 37°C than at 23°C, 28°C or 42°C with colonies being app. one third larger in diameter. At 28°C, growth of isolate IO-23 was completely inhibited for up to 3 d on LB agar containing 50 ug/ml ampicillin, 50 ug/ml kanamycin or 100 ug/ml streptomycin, whereas growth was not impaired by tetracycline concentrations up to 50ug/ml and was slightly and considerably reduced at 100 ug/ml and 200 ug/ml, respectively.

### Molecular taxonomy

PCR amplification and DNA sequencing on both strands led to the generation of confirmed consensus sequences for the different marker genes comprising in length 1,342 bp (16S rRNA), 615 bp (*fusA*), 798 bp (*gyrB*), 912 bp (*ileS*), 732 bp (*lepA*), and 408 bp (*leuS*). Sequence data have been submitted to the Genbank database under accession numbers MH553344-MH553351 and MH561863-MH561902. Pairwise consensus sequence similarities among Tunisian isolates as calculated from a p-distance matrix ranged from identity to 99.8% (*fusA*, *leuS)*, 99.7% (16S rRNA), or 99.6% *(ileS*), whereas amplified *gyrB* and *lepA* marker sequences from all isolates were identical. From isolates IO-6 and IO-20, identical sequences were amplified for all six markers. Moreover, two groups of isolates, namely i) IO-19 and IO-38, as well as ii) IO-23, IO-27, and IO-28, carried identical 16S rRNA, but different protein-encoding genes, whereas amplifications from isolates IO-23, IO-24, and IO-38 gave rise to identical protein-encoding, but different 16S rRNA gene sequences. When used as query in a search for highly similar Genbank database entries, all consensus sequences identified as best hit the respective orthologous gene from a *Providencia* bacterium.

### 16S rRNA gene based phylogenetic reconstruction

**Fig 1:**
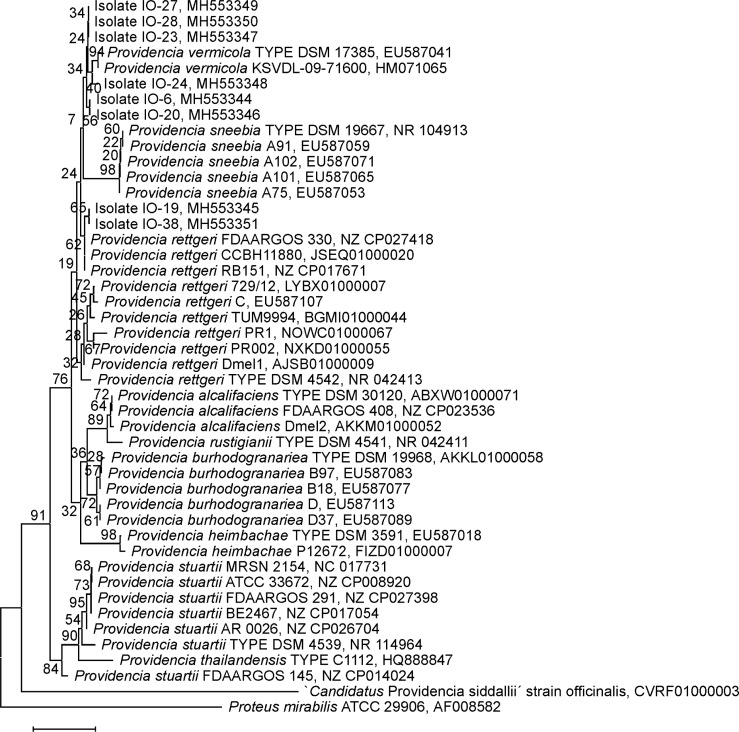
16S rRNA gene NJ tree. Neighbor Joining phylogeny of *Providencia* bacteria as reconstructed from 16S ribosomal RNA encoding sequences. Terminal branches are labelled by genus, species and strain designations as well as GenBank accession numbers. Numbers on branches indicate bootstrap support values. The size bar corresponds to 1% sequence divergence. An orthologous sequence from the closely related bacterium *Proteus mirabilis* has been used as outgroup.

For phylogenetic reconstruction from 16S rRNA encoding sequences, partial sequences from 245 bacteria assigned to the genus *Providencia* were identified in the GenBank database and retained as reference sequences. In the phylogenies reconstructed from this full set of sequence data (S8 Fig) and from a more interpretation relevant subset comprising 37 reference sequences (Fig 1, S1 Fig), the Tunisian isolates were dispersed across a broad, ill-supported clade comprising bacteria assigned to the species *P*. *rettgeri*, *P*. *vermicola*, *P*. *sneebia*, *and P*. *huaxiensis* sp. nov.. Average pairwise sequence similarities of Tunisian isolates to the type strains *P*. *rettgeri* DSM 4542T, *P*. *vermicola* DSM 17385T, *P*. *sneebia* DSM 19967T, and *P*. *huaxiensis* WCHPr000369T were, respectively, 99.6%, 99.7%, 99.2% and 99.4%, whereas the six pairs of 16S rRNA genes of these four type strains have between 99.0% and 99.6% (*P*. *rettgeri*—*P*. *huaxiensis*) of their sequences in common (Table 2). The isolates under study appeared, therefore, i) mostly closely related to the species *P*. *rettgeri*, *P*. *vermicola* and *P*. *huaxiensis* and ii) as closely related as these are to each other. The phylogenetic distance to all other *Providencia* species was larger with average pairwise sequence similarities to the respective type strains ranging from 99.2% (*P*. *sneebia* DSM 19967T) to 98.1% (*P*. *thailandiensis* C1112T) and 94.6% (`*Cand*. Providencia siddallii´) (Table 2).

**Table 2:** Pairwise sequence similarity percentages.

	Pent	PretB	PretT	PverT	PalcT	PburT	PheiT	PrusT	PsneT	PstuT	PthaT	PhuaT	PsidC	16S rRNA
*P*. *entomophila* (Pent)		99.8	99.6	99.7	99.0	99.1	98.9	98.9	99.2	98.6	98.1	99.4	94.6	Pent
*P*. *rettgeri* “clade B” (PretB)	99.8		99.6	99.8	99.1	99.2	99.1	98.9	99.2	98.7	98.3	99.3	94.7	PretB
*P*. *rettgeri* TYPE (PretT)	87.9	87.9		99.5	99.0	99.0	98.8	98.8	99.0	98.6	98.1	99.6	94.6	PretT
*P*. *vermicola* TYPE (PverT)	87.2	87.3	87.2		98.8	99.2	98.9	98.9	99.1	98.4	98.4	99.2	93.5	PverT
*P*. *alcalifaciens* TYPE (PalcT)	84.0	84.1	84.7	85.0		99.3	98.6	99.6	98.8	98.4	97.9	99.1	94.3	PalcT
*P*. *burhodogranariea* TYPE (PburT)	83.5	83.4	83.2	82.9	82.4		99.2	99.3	99.0	98.4	98.0	98.7	94.3	PburT
*P*. *heimbachae* TYPE (PheiT)	83.2	83.3	83.4	83.4	82.6	82.9		98.4	98.5	98.0	98.0	98.5	93.4	PheiT
*P*. *rustigiani* TYPE (PrusT)	85.0	85.0	84.9	84.9	87.3	83.8	84.7		98.6	98.1	97.7	98.9	94.1	PrusT
*P*. *sneebia* TYPE (PsneT)	82.4	82.4	83.0	82.5	82.2	84.8	82.6	84.2		98.4	98.1	99.0	94.2	PsneT
*P*. *stuartii* TYPE (PstuT)	82.8	82.9	82.8	83.2	83.8	84.1	82.6	89.4	84.0		99.1	98.5	94.1	PstuT
*P*. *thailandensis* TYPE (PthaT)	86.1	86.1	87.0	86.9	87.1	86.7	86.1	87.5	87.1	89.6		98.1	93.8	PthaT
*P*. *huaxiensis* TYPE (PhuaT)	88.2	88.2	92.9	81.1	84.6	83.5	84.1	85.3	83.1	82.9	86.4		94.3	PhuaT
`*Cand*. P. siddallii´ (PsidC)	n.d.	n.d.	n.d.	n.d.	n.d.	n.d.	n.d.	n.d.	n.d.	n.d.	n.d.	n.d.		PsidC
**concatenated MLSA markers**	Pent	PretB	PretT	PverT	PalcT	PburT	PheiT	PrusT	PsneT	PstuT	PthaT	PhuaT	PsidC	

Pairwise (average) nucleotide sequence similarity percentages as calculated from a p-distance matrix for the16S rRNA gene and concatenated MLSA markers from *Providencia* bacteria. Values calculated from 16S ribosomal RNA encoding sequences are displayed in the upper right-hand, those from concatenated MLSA marker sequences in the lower left-hand part of the table. Deviations from the mean value found for averaged similarities (not shown) have in no case been superior to 0.1%. Not determined values are marked “n.d.”.

### MLSA marker based phylogenetic reconstruction

For the five protein-encoding MLSA markers, a total of 58 (*fusA*), 59 (*gyrB*), 35 (*ileS*), 41 (*lepA*), and 46 (*leuS*) orthologous genes were identified in the GenBank database and retained as reference sequences for phylogenetic reconstruction. Pairwise nucleotide sequence similarities of these identified Genbank entries with respect to the corresponding marker from Tunisian isolate IO-19 ranged down to 89% (*fusA*), 83% (*gyrB*), 81% (*ileS*, *lepA*), and 80% (*leuS*). A subset of these data was used to generate a concatenation of the five marker sequences for a total of 38 *Providencia* reference strains.

**Fig 2:**
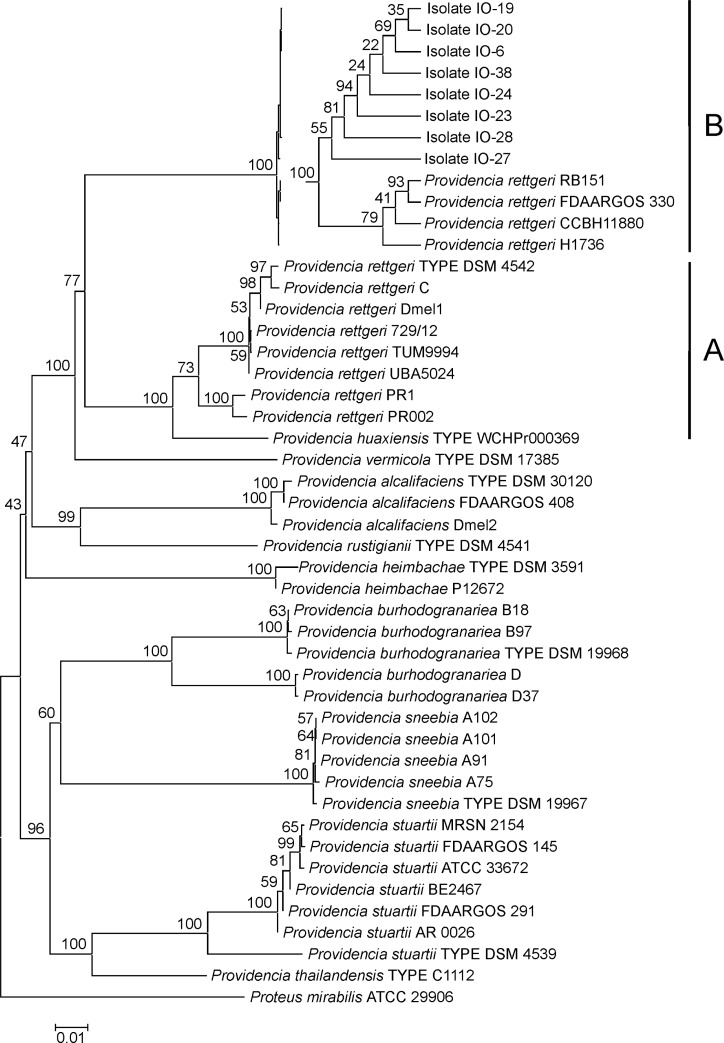
Concatenated MLSA marker NJ tree. Neighbor Joining (NJ) phylogeny of *Providencia* bacteria as reconstructed from concatenated MLSA marker nucleotide sequences. Terminal branches are labelled by genus, species and strain designations. GenBank accession numbers are given in the single gene trees, see S3–S7 Figs. Numbers on branches indicate bootstrap support values. The size bar corresponds to 1% sequence divergence along phylogram branches. Clades A and B referred to in the text have been indicated at the right margin; clade B comprising the Tunisian isolates has been expanded into a cladogram for better resolution. Concatenated orthologous sequences from the closely related bacterium *Proteus mirabilis* have been used as outgroup.

In the Neighbor Joining phylogeny reconstructed from these concatenated sequence data (Fig 2), the eight Tunisian isolates were located in an optimally (100%) bootstrap supported branch together with all *P*. *rettgeri* reference strains and the only available *P*. *vermicola* and *P*. *huaxiensis* references, i.e. the respective specific type strains DSM 17385T and WCHPr000369T. Groups of reference strains representing the further *Providencia* species, and in particular those known to comprise isolates stemming from or being pathogenic to fruit flies as *P*. *sneebia*, *P*. *burhodogranariea*, and *P*. *alcalifaciens*, formed distinct clades that in turn received 100% bootstrap support.

Within the branch comprising the Tunisian isolates, the *P*. *vermicola* type strain was placed in a sister clade position to two optimally bootstrap supported clades. One of these (termed “clade A”) comprised the majority of *P*. *rettgeri* reference strains including the specific type strain DSM 4542T together with the *P*. *huaxiensis* type strain, whereas the other (termed “clade B”) contained the eight Tunisian isolates together with four further strains assigned to *P*. *rettgeri*. These topological features were–with slightly different bootstrapping support values–reproduced in the Maximum Likelihood phylogeny generated from the same data set (S2 Fig). Moreover, localization of the Tunisian isolates in each of the five single MLSA marker phylogenies (S3–S7 Figs) was largely consistent with the picture emerging from the concatenated data set, thereby excluding that a single, more informative marker–potentially having been acquired by lateral gene transfer–overrode the cumulative phylogenetic signal from the other four genes.

When the concatenated marker sequences of the eight Tunisian isolates were compared to those of the reference strains representing *Providencia* species, average pairwise sequence similarities to the *P*. *rettgeri*, *P*. *huaxiensis*, and *P*. *vermicola* type strains were 87.9%, 88.2%, and 87.2%, respectively, whereas similarities to the further–presumably more distantly related—*Providencia* species ranged from 86.1% (*P*. *thailandiensis* C1112T) to 82.4% (*P*. *sneebia* DSM 19967T) (Table 2). These values were very much in line with sequence similarities between type strains of the different *Providencia* species as, e.g., *P*. *rettgeri* DSM 454T and *P*. *vermicola* DSM 17385T (87.2%). The Tunisian isolates studied appeared, therefore, as closely or distantly related to the species *P*. *rettgeri*, *P*. *huaxiensis*, and *P*. *vermicola–*as represented by the respective type strains—as these were to each other, and more distantly related to the further *Providencia* species. As a notable exception, the sequence similarity of 92.9% calculated for the type strain pair *P*. *rettgeri* DSM 454T and *P*. *huaxiensis* WCHPr000369T indicated a comparatively closer relationship of the recently introduced new species to *P*. *rettgeri*.

However, when the Tunisian isolates were compared not only to the *P*. *rettgeri* type strain, but also to the further reference strains from this species, average pairwise similarities of concatenated MLSA marker sequences were strongly biased between the *P*. *rettgeri* strains and the *P*. *huaxiensis* type strain comprised in clade A of the NJ phylogeny (Fig 2) with values ranging from 87.9% to 88.2% as opposed to 99.8% with respect to the four strains comprised in clade B. Moreover, the average sequence similarity of the strains in clade B to the *P*. *rettgeri* and *P*. *huaxiensis* type strains was as low as 87.9% and 88.2%, respectively, and still lower when compared to further *Providencia* type strains (Table 2). Thus, supposed *P*. *rettgeri* strains in clade B and the Tunisian *Providencia* isolates appeared closely related to each other, but only distantly related to all type strains representing the currently recognized *Providencia* species, including *P*. *rettgeri*, as well as to the recently proposed new species *P*. *huaxiensis*.

### DNA-DNA hybridization

DNA–DNA hybridization was performed between isolate IO-23 and *P*. *rettgeri* DSM 4542T as the most closely related type strain with respect to concatenated MLSA marker sequence distances. The average reassociation value determined was 25.4% (i.e. the arithmetic mean of 23.0%and 27.7% as obtained from two separate measurements).

### Metabolic profiling

**Fig 3:**
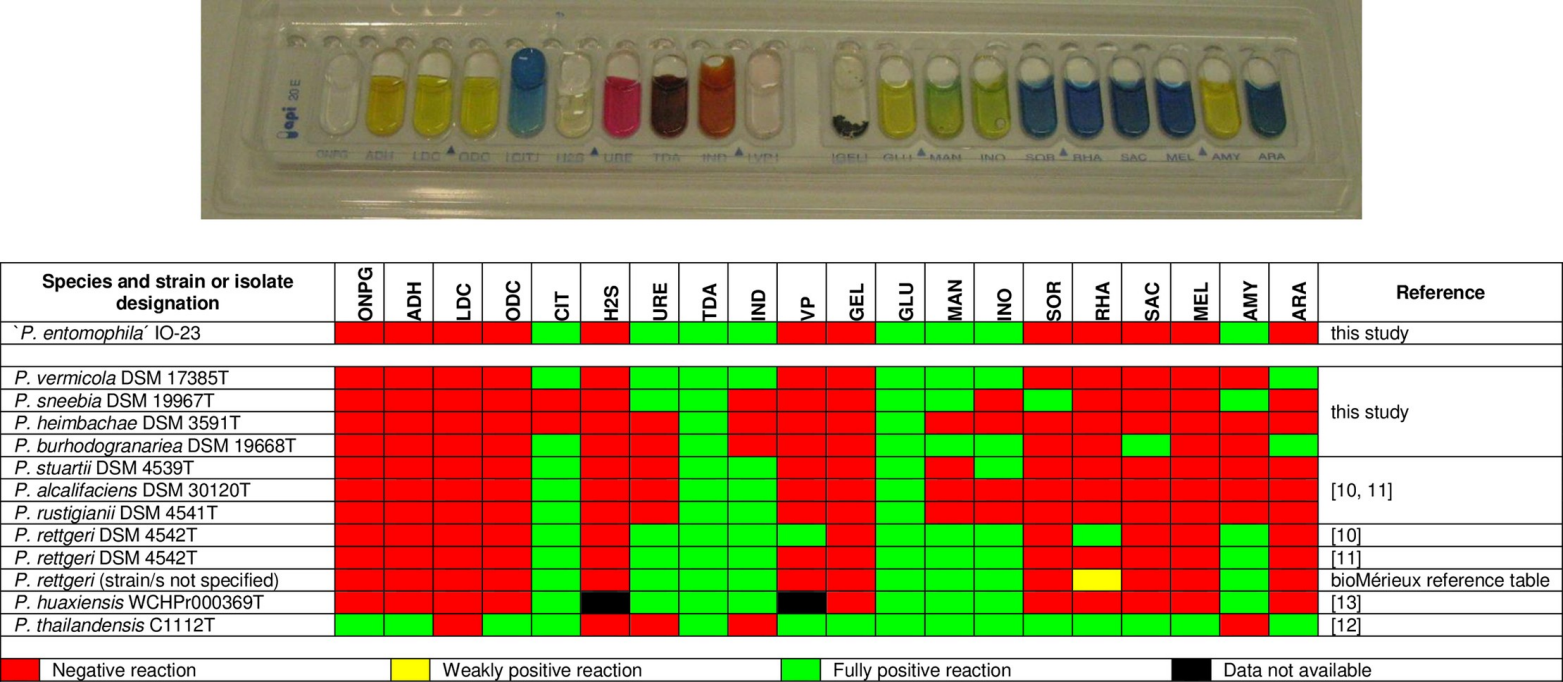
Comparison of metabolic profiles of *Providencia* bacteria as determined by the API 20E test system. **Upper part:** Picture of an API20E test result obtained with isolate IO-23 after 48 h of incubation. Reactions are in the same order as indicated in the table below. **Lower part:** Graphic representation (heat chart) of API20E test results for Tunisian isolate IO-23 and type strains of further *Providencia* species. Activities indicated in the top row are as follows: beta-galactosidase (ONPG), L-arginine dihydrolase (ADH), L-lysine decarboxylase (LDC), L-ornithine decarboxylase (ODC), citrate utilization (CIT), H_2_S production (H2S), urease (URE), L-tryptophane desaminase (TDA), indole production (IND), acetoin production (VP), gelatinase (GEL), D-glucose fermentation/oxidation (GLU), D-mannitol fermentation/oxidation (MAN), inositol fermentation/oxidation (INO), D-sorbitol fermentation/oxidation (SOR), L-rhamnose fermentation/oxidation (RHA), D-sacchose/sucrose fermentation/oxidation (SAC), D-melibiose fermentation/oxidation (MEL), amygdalin fermentation/oxidation (AMY), L-arabinose fermentation/oxidation (ARA).

The metabolic profiles of the Tunisian isolates were identical to that of isolate IO-23. Isolates were found able to utilize citrate, D-glucose, D-mannitol, inositol and amygdalin, to produce indole from L-tryptophane, and displayed urease and L-tryptophane desaminase activities. All other reactions assayed were negative (Fig 3). The metabolic profile of the Tunisian isolates thereby differed by two to six traits from those of the *Providencia* reference strains assayed, i.e. *P*. *heimbachae* DSM 3591T, *P*. *vermicola* DSM 17385T, *P*. *sneebia* DSM 19967T, and *P*. *burhodogranariea* DSM 19668T (Fig 3).

### Bioassays

Bioassays against the olive fly were performed with bacterial isolates IO-20, IO-23, IO-27, and IO-28 that had been isolated from this host or from its empty galleries found inside damaged fruit (Table 1).

**Fig 4:**
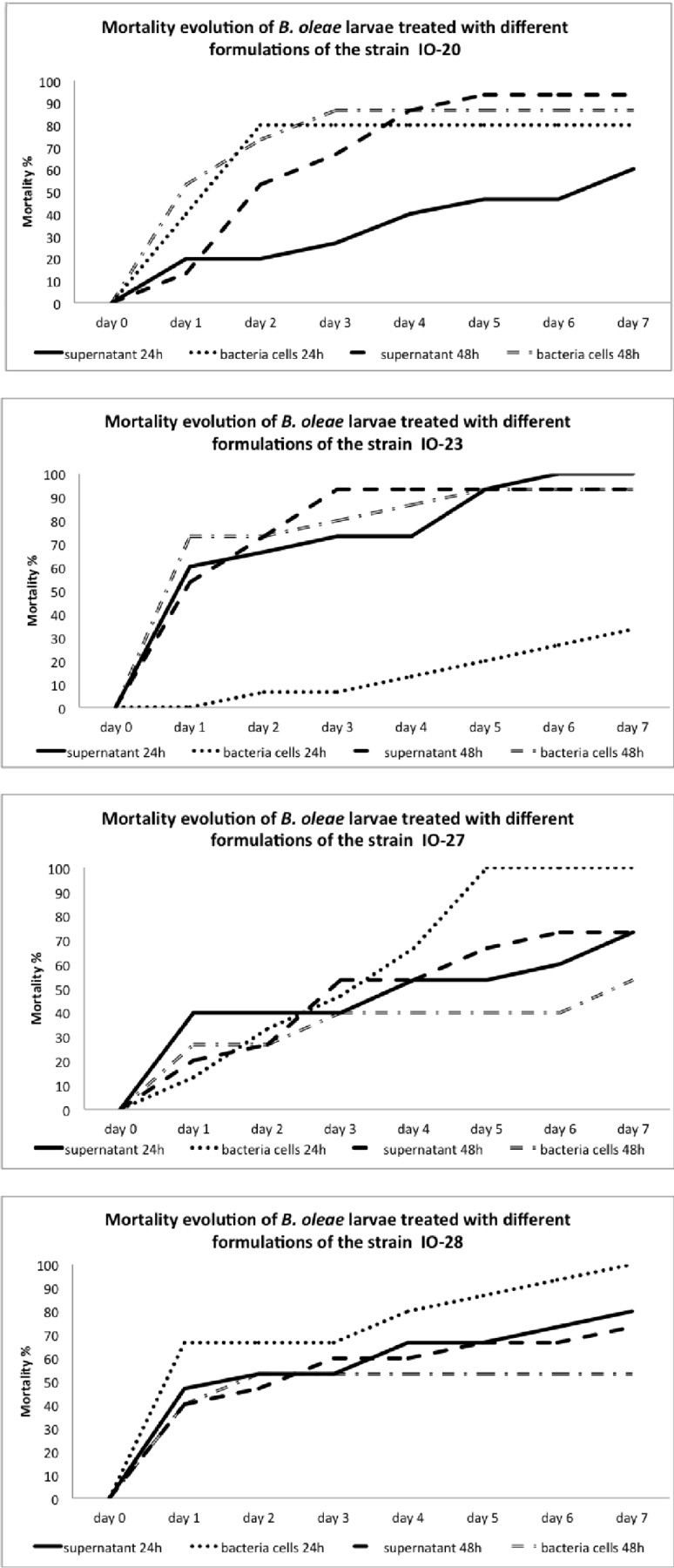
Virulence bioassay: Mortality profiles. Accumulated mortality profiles (in %) for virulence bioassays with four bacterial isolates against adult olive flies. Different line drawings correspond to the inoculation methods described under materials and methods.

The majority of the tested combinations of bacterial strains and treatment conditions were found to cause a significant increase in olive fly mortality as compared to that of the control (Fig 4, Table 3). When treated with culture supernatant (24h and 48h) of all strains tested and with bacterial cells of isolates IO-20, IO-27, and IO-28, olive fly adults showed mortality rates significantly far more important than those obtained with the control. As exception from this general pattern, the increase in mortality caused by cells from a 24 h culture of strain IO-23 was statistically not significant. However, when cells from a 48 h culture where applied, the mortalities caused by isolates IO-20 and IO-23 were not only significantly higher than those of the control, but also than those caused by cells from analogous cultures of isolates IO-27 and IO-28. Statistical analysis indicates the absence of any significant difference between the different types of treatments for strains IO-20 and IO-28. Concerning strain IO-23, it appears that the treatment with bacterial cells (24h) gives statistically the lowest mortality rate (33.33%). However, for the strain IO-27, it was noted that the treatment with the bacteria cells (24h) gave rise to 100% of mortality, followed by treatments with the supernatants (24 and 48h) which caused an intermediate mortality, and finally by treatment with bacteria cells (48h) which produced the lowest mortality (around 53.33%).

**Table 3:** Virulence bioassay: Accumulated mortalities and Tukey test.

Treatment	IO-20	IO-23	IO-27	IO-28	Control	
**Supernatant (24h)**	60 ± 20 Aa	100 ± 0 Aa	73.33 ± 23.09 Aab	80 ± 20 Aa	20 ± 0 B	df = 4; F = 10,000; P = 0.002.
**Bacterial cells (24h)**	80 ± 20 Aa	33.33 ± 23.09 Bb	100 ± 0 Aa	100 ± 0 Aa	20 ± 0 B	df = 4; F = 22,857; P = 0.0001.
**Supernatant (48h)**	100 ± 0 Aa	93.33 ± 11.55 Aa	73.33 ± 11.55 Aab	73.33 ± 23.09 Aa	20 ± 0 B	df = 4; F = 18,500; P = 0.0001.
**Bacterial cells (48h)**	86.66 ± 23.09 Aa	93.33 ± 11.55 Aa	53.33 ± 11.55 ABb	53.33 ± 23.09 ABa	20 ± 0 B	df = 4; F = 9,850; P = 0.002.
	df = 3; F = 1.212; P = 0.374.	df = 3; F = 9.848; P = 0.005.	df = 3; F = 4.934; P = 0.032.	df = 3; F = 3.094; P = 0.090.	*	

Mortality rates (in %) determined for *Providencia* isolates assayed against adults of the olive fly. Values followed by the same letter are not statistically different using mean comparisons (Tukey test; P<0.05) angular transformed data. Capital letters represent comparisons within a row and lower case letters comparisons within a column. The asterisk “*” indicates that statistical analysis is not feasible (due to equal percentages for all data).

## Discussion

Within the framework of previous research activities aiming to explore the bacterial and fungal microflora associated with three major olive tree pests, eight bacterial cultures isolated from different locations in central Tunisia had been identified as members of the genus *Providencia* by preliminary comparison of 16S rRNA gene sequences. This had been the first report of the isolation of *Providencia* bacteria from the olive fly, the olive moth, and the olive psyllid [4]. In the present study, four of these isolates were shown to increase mortality of adult olive flies. If independently corroborated, entomopathogenicity would make these bacteria an interesting object of biocontrol research with respect to a highly relevant group of pest insects as are tephritid or drosophilid flies.

*Providencia* bacteria had previously been found associated with Dipteran insects as the Mexican fruit fly, *Anastrepha ludens* (Loew) (Diptera; Tephritidae) [15], wild-caught individuals of the vinegar fly, *Drosophila melanogaster* (Meigen) (Diptera; Drosophilidae) [11], and a laboratory strain of the Mediterranean fruit fly, *Ceratitis capitata* (Wiedemann) (Diptera; Tephritidae) [16], but not with the olive fly. Moreover, *D*. *melanogaster* and *C*. *capitata* derived strains assigned to the taxonomic species *P*. *alcalifaciens*, *P*. *sneebia* or *P*. *rettgeri* had been shown to display pronounced virulence against their respective hosts [16, 17], whereas strains belonging to the species *P*. *vermicola* had been found associated with juveniles of entomopathogenic nematodes and were demonstrated to cause mortality of Lepidopteran insects independent from nematode-association [10, 29, 30]. It is to date largely unexplored if there is specific adaptation of different *Providencia* species to their respective fruit fly hosts.

16S rRNA based molecular taxonomy identified the new bacterial isolates as most closely related to the recognized *Providencia* species *P*. *sneebia*, *P*. *rettgeri* or *P*. *vermicola* as well as the recently proposed new species *P*. *huaxiensis*. However, species assignment by both phylogenetic reconstruction and p-distance analysis remained inconclusive on the basis of 16S rRNA sequences alone. Phylogenetic reconstruction using an MLSA scheme consisting of five protein-encoding markers firstly corroborated the results from 16S rRNA gene phylogenies while responding to more severe confidence criteria.

Moreover, detailed analysis of pairwise sequence p-distances demonstrated that Tunisian isolates i) were closely related to each other and thus belonged to the same taxonomic species and ii) were as distantly related to the type strains representing all *Providencia* species including *P*. *huaxiensis* sp. nov. as these are from each other, i.e. Tunisian isolates should most appropriately be assigned to a new *Providencia* species. In particular, molecular taxonomic analysis made it clear that the Tunisian isolates were distinct at the taxonomic species level from *Providencia* isolates that had previously been described as associated with or pathogenic to insects, in particular fruit flies [10, 11, 15, 16].

However, the emerging picture appeared complicated as several bacterial strains that had recently been described as *P*. *rettgeri* [31–33] together with the Tunisian isolates formed a well-supported and tight group of bacteria (marked “clade B” in Fig 2 and S3 Fig), while the majority of *P*. *rettgeri* strains including the type strain DSM 4542T gave rise to a respective sister clade (marked “clade A”). Comparative analysis of pairwise MLSA marker sequence distances across the genus *Providencia* i) clearly demonstrated that the clade B strains in question should not any further be assigned to the species *P*. *rettgeri* and ii) was fully consistent with the organization of these strains and the Tunisian isolates in the same *Providencia* species (Table 2).

The *Providencia* strains comprised in this potentially new species formed a compact, presumably monophyletic group with concatenated MLSA marker based pairwise sequence similarities not below 99.8%. Given the highly diverse materials of origin, i.e. different types of human clinical samples in addition to those mentioned in Table 1, and geographic origins of these strains, namely Brazil, Colombia, Israel, and the U.S.A. in addition to Tunisia, the group’s genetic compactness can hardly be explained by biased sampling due to geographic clustering or host adaptation.

When assayed with the API 20E identification system for enterobacteria, the bacterial isolates from Tunisia reproducibly gave rise to a unique metabolic profile that unambiguously discriminated them by two to six metabolic traits from most of the currently recognized *Providencia* species (Fig 3). However, comparison to published metabolic profiles for *P*. *rettgeri* and *P*.*huaxiensis* bacteria was less clear as these were not fully consistent with respect to several of the enzymatic reactions included in the API 20E test strips as, e.g., acetoin production and L-rhamnose utilization tests. In most cases, these differences might be the consequence of variations in experimental settings as reaction temperature and duration employed by different authors [7, 10, 11, 13, 34]. All Tunisian isolates consistently gave negative results for both the acetoin production and L-rhamnose utilization test even after prolonged (48h) incubation (Fig 3). Lack of acetoin production and L-rhamnose utilization had been reported by [11] for the *P*. *rettgeri* type strain, whereas [10] had found that this strain produced acetoin and utilized L-rhamnose. According to both [34] and the reference table issued by the manufacturer of the API 20E test system, the species *P*. *rettgeri* displayed an intermediate or weak ability to utilize L-rhamnose, the standardized test giving a positive result only after prolonged incubation. L-rhamnose utilization, therefore, most likely constitutes a metabolic difference between the Tunisian isolates and the taxonomic species *P*. *rettgeri*. However, discrimination from *P*. *rettgeri* and *P*. *huaxiensis* sp. nov. was not as unambiguous as from the further recognized *Providencia* species.

Earlier genomic DNA-DNA hybridization studies had reported reassociation values ranging from 13% to 49% for pairs of strains belonging to different *Providencia* species [7, 8, 10, 11]. The value of 25% determined for the Tunisian isolate IO-23 and *P*. *rettgeri* DSM 4542T falls well into this range and far below the 70% threshold considered by [35] for assignment of two strains to the same taxonomic species. DNA-DNA hybridization results therefore clearly demonstrated that Tunisian isolate IO-23 does not belong to the phylogenetically and metabolically most closely related of the currently recognized species within the genus *Providencia*, i.e. *P*. *rettgeri*.

## Conclusions

Combined evidence from metabolic profiling studies, MLSA-based phylogenetic reconstruction and genomic DNA-DNA hybridization demonstrated that a set of bacterial isolates associated with pest insects in olive orchards in Tunisia represents a new taxonomic species within the bacterial genus *Providencia*. With reference to the association with insects, the species epithet “*entomophila*” is proposed for naming the new taxon.

### Description of *Providencia entomophila* sp. nov.

*Providencia entomophila* (en.to.mo´phi.la. N.L. pref. *entomo–*from Gr. vb. *én-témno* to cut in, to engrave, used to refer to insects due to the segmented body structure; N.L. suff.–*phila* from Gr. pref. *philo-* loving, liking; N.L. adj. *entomophila* insect liking) readily cultivable Gram-negative, rod-shaped bacterium. Colonies grown on LB agar for 24/48 h at 28°C are up to 4 mm in diameter, cream coloured, opaque, glossy, smooth, and convex in appearance. Growth occurs faster at 32°C and 37°C than at 28°C or 42°C. Colonies are cream coloured after 24h of growth on MacConkey agar and on LB agar containing X-Gal. The bacterium is resistant to up to 100 μg/ml tetracycline on LB plates, utilizes citrate, D-glucose, D-mannitol, inositol and amygdalin, produces indole from L-tryptophane, and displayes urease and L-tryptophane desaminase activities.The type strain IO-23 was isolated from dead adults of *Bactrocera oleae* from an olive orchard in El Amra, Sfax, Tunisia.

## Supporting information

S1 Table Oligonucleotide primers and reaction-specific PCR parameters. (DOC)

S1 Fig 16S rRNA gene ML tree. Maximum Likelihood (ML) phylogeny of *Providencia* bacteria as reconstructed from 16S ribosomal RNA encoding sequences. Terminal branches are labelled by genus, species and strain designations as well as GenBank accession numbers. Numbers on branches indicate bootstrap support values. The size bar corresponds to 1% sequence divergence. The branch representing `*Candidatus* Providencia siddallii´ (shown as dashed line) has been shortened to 25% of its original length to facilitate graphical presentation. An orthologous sequence from the closely related bacterium *Proteus mirabilis* has been used as outgroup.
(PDF)

S2 Fig Concatenated MLSA marker ML tree. Maximum Likelihood (ML) phylogeny of *Providencia* bacteria as reconstructed from concatenated MLSA marker nucleotide sequences. Terminal branches are labelled by genus, species and strain designations. GenBank accession numbers are given in the single gene trees, see S4–S8 Figs. Numbers on branches indicate bootstrap support values. The size bar corresponds to 5% sequence divergence along phylogram branches. Clades A and B referred to in the text have been indicated at the right margin; clade B comprising the Tunisian isolates has been expanded into a cladogram for better resolution. Concatenated orthologous sequences from the closely related bacterium *Proteus mirabilis* have been used as outgroup.
(PDF)

S3 Fig fusA gene NJ tree. Neighbor Joining (NJ) phylogeny of *Providencia* bacteria as reconstructed from *fusA* gene sequences. Terminal branches are labelled by genus, species and strain designations as well as GenBank accession numbers. Numbers on branches indicate bootstrap support values. The size bar corresponds to 1% sequence divergence. An orthologous sequence from the closely related bacterium *Proteus mirabilis* has been used as outgroup.
(PDF)

S4 Fig gyrB gene NJ tree. Neighbor Joining (NJ) phylogeny of *Providencia* bacteria as reconstructed from *gyrB* gene sequences. Terminal branches are labelled by genus, species and strain designations as well as GenBank accession numbers. Numbers on branches indicate bootstrap support values. The size bar corresponds to 1% sequence divergence. An orthologous sequence from the closely related bacterium *Proteus mirabilis* has been used as outgroup.
(PDF)

S5 Fig ileS gene NJ tree. Neighbor Joining (NJ) phylogeny of *Providencia* bacteria as reconstructed from *ileS* gene sequences. Terminal branches are labelled by genus, species and strain designations as well as GenBank accession numbers. Numbers on branches indicate bootstrap support values. The size bar corresponds to 1% sequence divergence. An orthologous sequence from the closely related bacterium *Proteus mirabilis* has been used as outgroup.
(PDF)

S6 Fig lepA gene NJ tree. Neighbor Joining (NJ) phylogeny of *Providencia* bacteria as reconstructed from *lepA* gene sequences. Terminal branches are labelled by genus, species and strain designations as well as GenBank accession numbers. Numbers on branches indicate bootstrap support values. The size bar corresponds to 1% sequence divergence. An orthologous sequence from the closely related bacterium *Proteus mirabilis* has been used as outgroup.
(PDF)

S7 Fig leuS gene NJ tree. Neighbor Joining (NJ) phylogeny of *Providencia* bacteria as reconstructed from *leuS* gene sequences. Terminal branches are labelled by genus, species and strain designations as well as GenBank accession numbers. Numbers on branches indicate bootstrap support values. The size bar corresponds to 1% sequence divergence. An orthologous sequence from the closely related bacterium *Proteus mirabilis* has been used as outgroup.
(PDF)

S8 Fig 16S rRNA gene full NJ tree. Neighbor Joining (NJ) phylogeny of *Providencia* bacteria as reconstructed from 16S ribosomal RNA encoding sequences. Terminal branches are labelled by GenBank accession numbers as well as genus, species and strain designations. Numbers on branches indicate bootstrap support values >80%. The size bar corresponds to 1% sequence divergence. An orthologous sequence from the closely related bacterium *Proteus mirabilis* has been used as outgroup.
(TIF)
