# Supplementary material for: Providencia entomophila sp. nov., a new bacterial species associated with major olive pests in Tunisia
Source: PLoS One. 2019 Oct 22;14(10):e0223943. doi: 10.1371/journal.pone.0223943 (PMC6805009; doi:10.1371/journal.pone.0223943)
Supplement: S1 Table — (DOC) [file pone.0223943.s001.doc]

**S1 Table. Oligonucleotide primers and reaction-specific PCR parameters.**

| **Primer Designation** | **Primer Sequence** | **Annealing temperature (°C)** | **Elongation**  **time**  **(sec)** | **Reference** |
| --- | --- | --- | --- | --- |
| fD1 | 5’-TGAAGAGTTTGATCCTGGCTCAG | 52 | 120 | Weisburg et al., (1991) |
| rP2 | 5’-CCTACGGCTACCTTGTTACGACTT |
| 522f | 5’-GTGCCAGCAGCCGCGGTAATAC | sequencing primer | |
| 760r | 5’-CCAGGGTATCTAATCCTGTTTGC | sequencing primer | |
| fusA-provF | 5’-GGACTGGATGGAGCAGGA | 50 | 60 | Juneja & Lazzaro (2009) |
| fusA-provR | 5’-TGCAGAACCACAGGTAACCA |
| fusA-F2 | 5’-CTGCATTCTGGTCTGGTATG | sequencing primer | | this study |
| fusA-R2 | 5’-TTCATCGCTCCACTTGATTG | sequencing primer | |
| gyrB-provF | 5’-TATCGGTGATACCGACGATGG | 50 | 120 | Juneja & Lazzaro (2009) |
| gyrB-provR | 5’-CGCARTTTATCTGGGTT |
| gyrB-F2 | 5’-AAGGTGTCACAGAATTCGAG | sequencing primer | | this study |
| gyrB-R2 | 5’-TAGGGTCTGGAACTTTCACG | sequencing primer | |
| ileS-provF | 5’-CCGATTGAACACAAAGTTGAA | 50 | 120 | Juneja & Lazzaro (2009) |
| ileS-provR | 5’-AGATCCCACCATGCTTGA |
| ileS-F2 | 5’-ACACCTTCATTAGTTATCTG | sequencing primer | | this study |
| ileS-R2 | 5’-TTTATGGCGCCAACAGCATG | sequencing primer | |
| lepA-provF | 5’-CCGTATTATTCAGATTTGTGGTG | 50 | 90 | Juneja & Lazzaro (2009) |
| lepA-provR | 5’-TGACTGGGAATAAACCTGCAT |
| lepA-F2 | 5’-ACTGTTATACCGCTATTGAG | sequencing primer | | this study |
| lepA-R2 | 5’-GCGGTCAATTCGCTTCGGTG | sequencing primer | |
| leuS-provF | 5’-TGCTGGCGYTGTGAYAC | 50 | 60 | Juneja & Lazzaro (2009) |
| leuS-provR | 5’-AAACACCCCAGTCACG |
| leuS-F2 | 5’-TTATGGGGGCGACTTACGTG | sequencing primer | | this study |
| leuS-R2 | 5’-TCAGAGAACCTTTATCCGTC | sequencing primer | |

**References**

Weisburg WG, Barns SM, Pelletier DA, Lane DJ. [16S ribosomal DNA amplification for phylogenetic study.](https://www.ncbi.nlm.nih.gov/pubmed/1987160) J Bacteriol. 1991;173: 697-703.

Juneja P, Lazzaro BP. *Providencia sneebia* *sp. nov.* and *Providencia burhodogranariea sp. nov.,* isolated from wild *Drosophila melanogaster*. Int J Syst Evol Microbiol. 2009;59: 1108–1111.
