# Supplementary material for: Providencia entomophila sp. nov., a new bacterial species associated with major olive pests in Tunisia
Source: PLoS One. 2019 Oct 22;14(10):e0223943. doi: 10.1371/journal.pone.0223943 (PMC6805009; doi:10.1371/journal.pone.0223943)
Supplement: S1 Fig — Maximum Likelihood (ML) phylogeny of Providencia bacteria as reconstructed from 16S ribosomal RNA encoding sequences. Terminal branches are labelled by genus, species and strain designations as well as GenBank accession numbers. Numbers on branches indicate bootstrap support values. The size bar corresponds to 1% sequence divergence. The branch representing `Candidatus Providencia siddallii´ (shown as dashed line) has been shortened to 25% of its original length to facilitate graphical presentation. An orthologous sequence from the closely related bacterium Proteus mirabilis has been used as outgroup. (PDF) [file pone.0223943.s002.pdf]

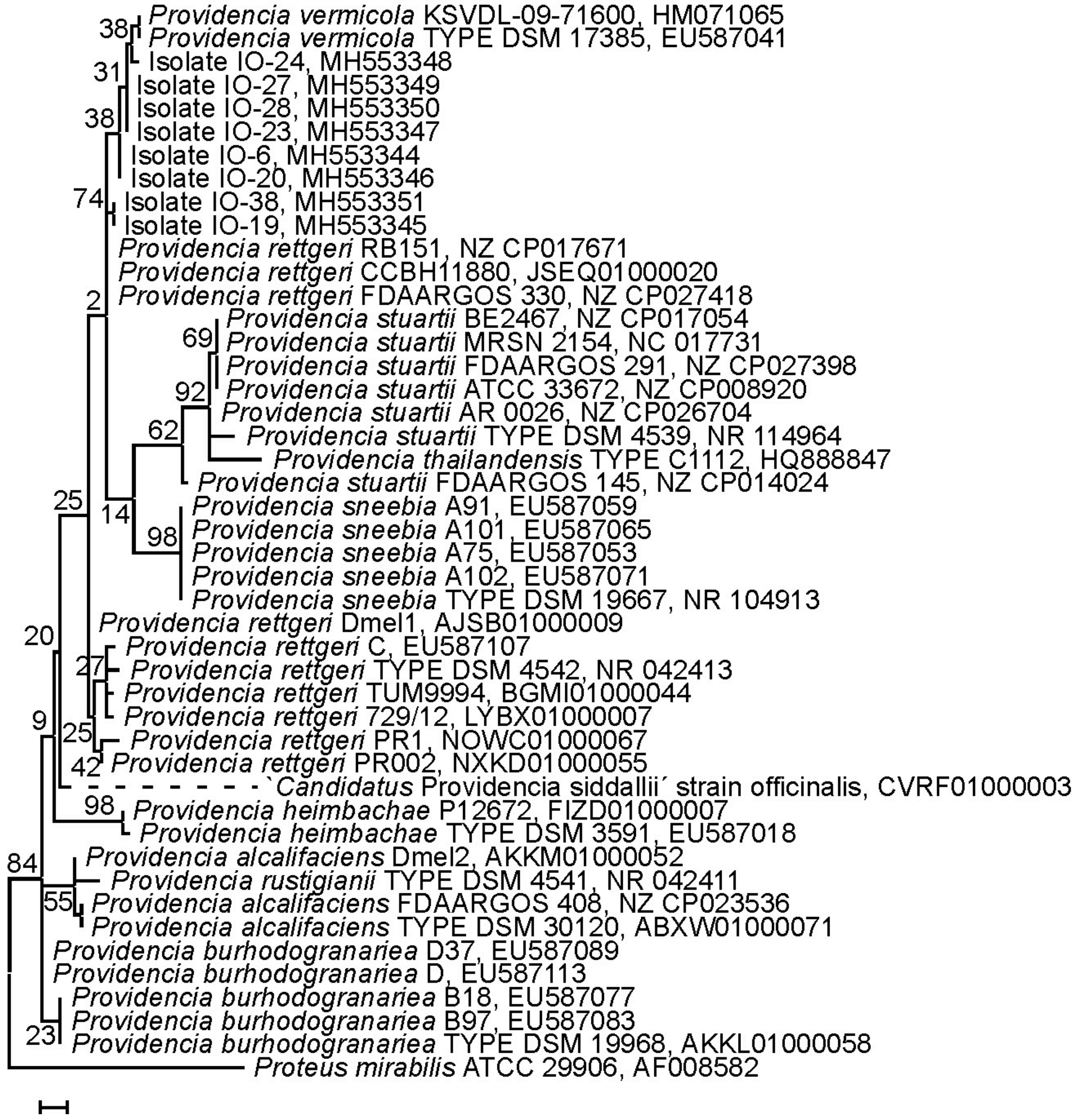

S1 Fig. 16S rRNA gene ML tree. Maximum Likelihood (ML) phylogeny of *Providencia* bacteria as reconstructed from 16S ribosomal RNA encoding sequences. Terminal branches are labelled by genus, species and strain designations as well as GenBank accession numbers. Numbers on branches indicate bootstrap support values. The size bar corresponds to 1 % sequence divergence. The branch representing `*Candidatus* Providencia siddallii´ (shown as dashed line) has been shortened to 25 % of its original length to facilitate graphical presentation . An orthologous sequence from the closely related bacterium *Proteus mirabilis* has been used as outgroup.
