# Supplementary material for: Providencia entomophila sp. nov., a new bacterial species associated with major olive pests in Tunisia
Source: PLoS One. 2019 Oct 22;14(10):e0223943. doi: 10.1371/journal.pone.0223943 (PMC6805009; doi:10.1371/journal.pone.0223943)
Supplement: S2 Fig — Maximum Likelihood (ML) phylogeny of Providencia bacteria as reconstructed from concatenated MLSA marker nucleotide sequences. Terminal branches are labelled by genus, species and strain designations. GenBank accession numbers are given in the single gene trees, see S4–S8 Figs. Numbers on branches indicate bootstrap support values. The size bar corresponds to 5% sequence divergence along phylogram branches. Clades A and B referred to in the text have been indicated at the right margin; clade B comprising the Tunisian isolates has been expanded into a cladogram for better resolution. Concatenated orthologous sequences from the closely related bacterium Proteus mirabilis have been used as outgroup. (PDF) [file pone.0223943.s003.pdf]

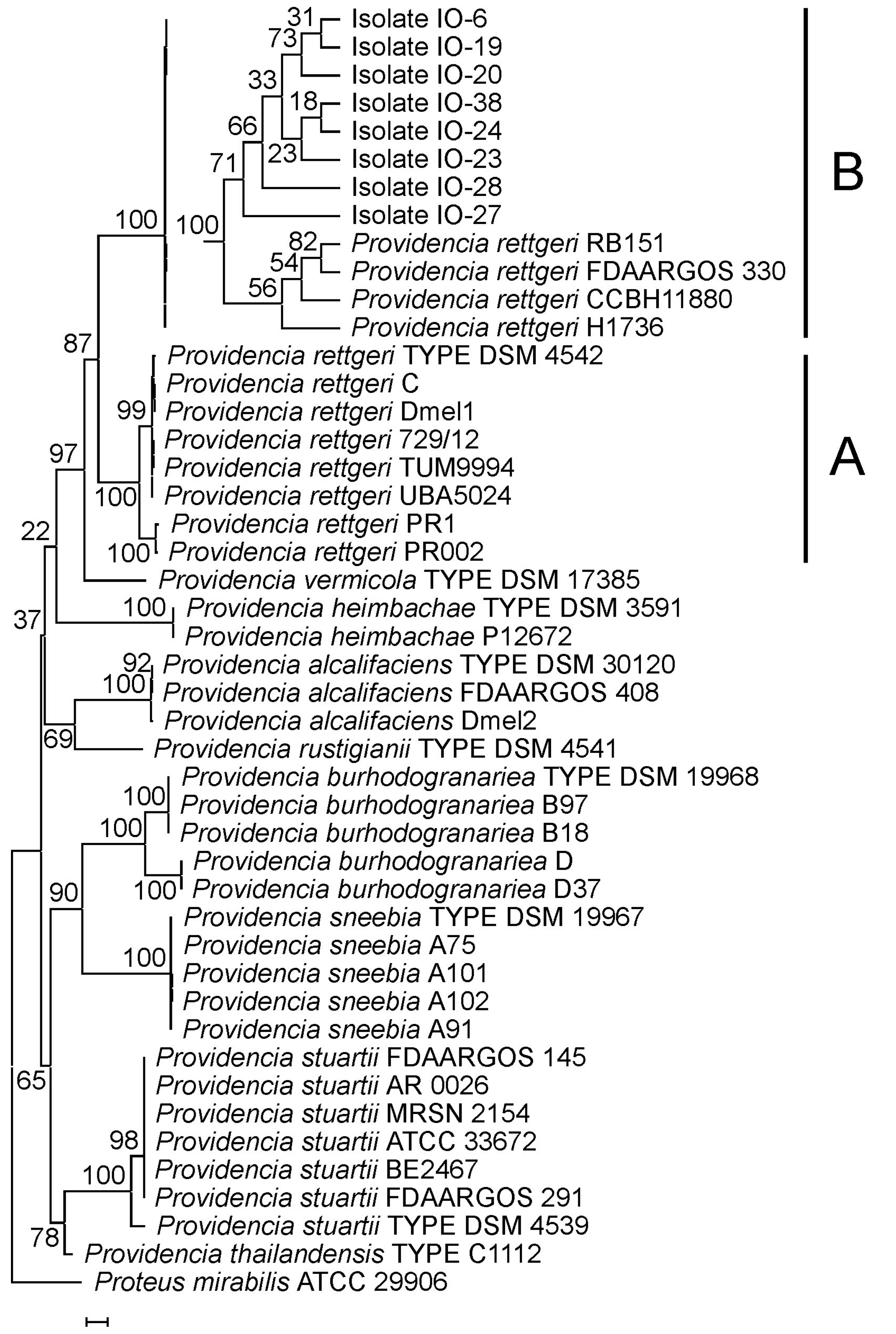

S2 Fig. Concatenated MLSA marker ML tree. Maximum Likelihood (ML) phylogeny of *Providencia* bacteria as reconstructed from concatenated MLSA marker nucleotide sequences. Terminal branches are labelled by genus, species and strain designations. GenBank accession numbers are given in the

single gene trees, see S3-S7 Figs. Numbers on branches indicate bootstrap support values. The size bar corresponds to 5 % sequence divergence along phylogram branches. Clades A and B referred to in the text have been indicated at the right margin; clade B comprising the Tunisian isolates has been expanded into a cladogram for better resolution. Concatenated orthologous sequences from the closely related bacterium *Proteus mirabilis* have been used as outgroup.
