# Supplementary material for: Providencia entomophila sp. nov., a new bacterial species associated with major olive pests in Tunisia
Source: PLoS One. 2019 Oct 22;14(10):e0223943. doi: 10.1371/journal.pone.0223943 (PMC6805009; doi:10.1371/journal.pone.0223943)
Supplement: S3 Fig — Neighbor Joining (NJ) phylogeny of Providencia bacteria as reconstructed from fusA gene sequences. Terminal branches are labelled by genus, species and strain designations as well as GenBank accession numbers. Numbers on branches indicate bootstrap support values. The size bar corresponds to 1% sequence divergence. An orthologous sequence from the closely related bacterium Proteus mirabilis has been used as outgroup. (PDF) [file pone.0223943.s004.pdf]

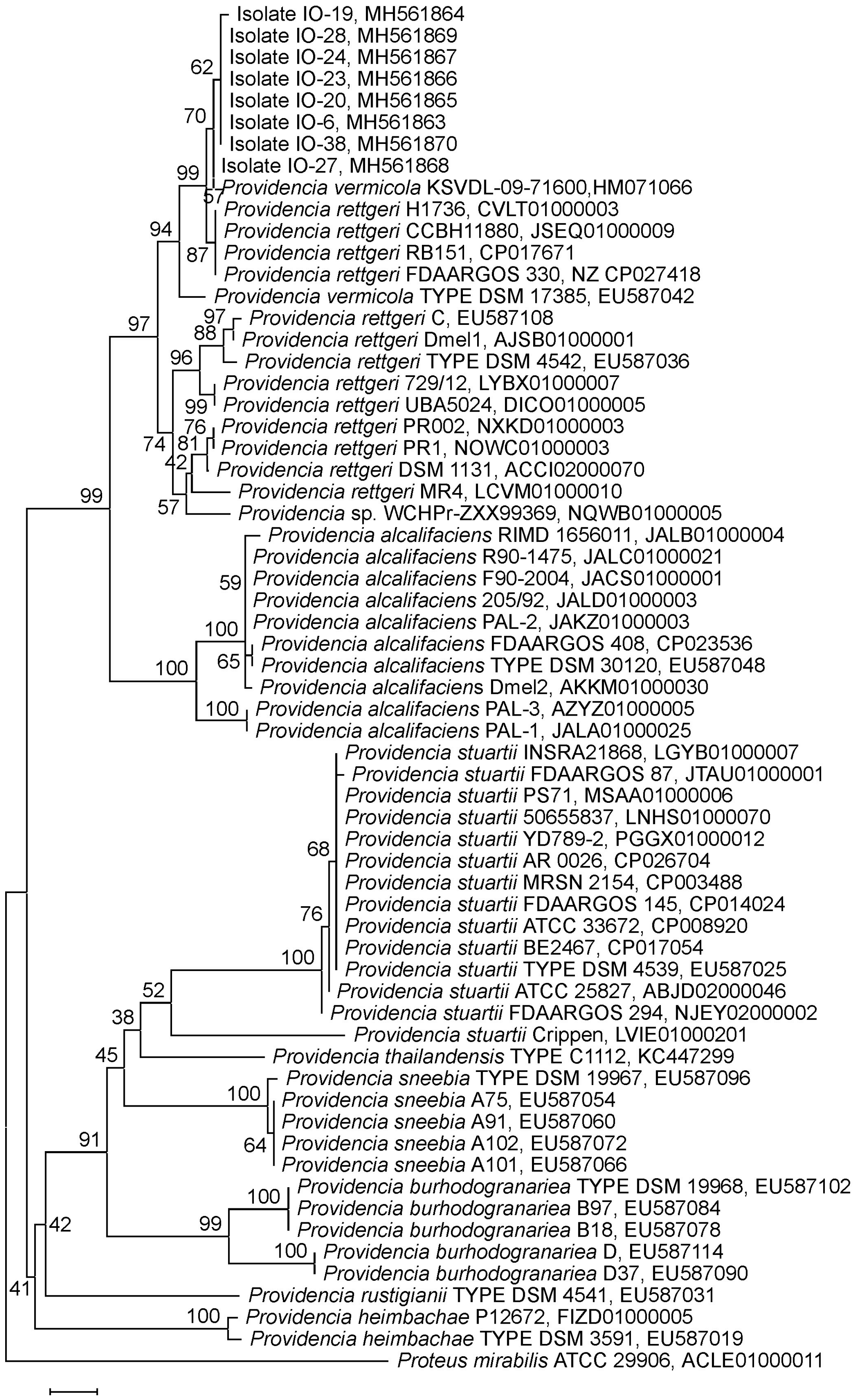

Isolate IO-19, MH561864
Isolate IO-28, MH561869
Isolate IO-24, MH561867
Isolate IO-23, MH561866
Isolate IO-20, MH561865
Isolate IO-6, MH561863
Isolate IO-38, MH561870
Isolate IO-27, MH561868
Providencia vermicola KSVDL-09-71600,HM071066
Providencia rettgeri H1736, CVLT01000003
Providencia rettgeri CCBH11880, JSEQ01000009
Providencia rettgeri RB151, CP017671
Providencia rettgeri FDAARGOS 330, NZ CP027418
Providencia vermicola TYPE DSM 17385, EU587042
Providencia rettgeri C, EU587108
Providencia rettgeri Dmel1, AJSB01000001
Providencia rettgeri TYPE DSM 4542, EU587036
Providencia rettgeri 729/12, LYBX01000007
Providencia rettgeri UBA5024, DICO01000005
Providencia rettgeri PR002, NXKD01000003
Providencia rettgeri PR1, NOWC01000003
Providencia rettgeri DSM 1131, ACCI02000070
Providencia rettgeri MR4, LCVM01000010
Providencia sp. WCHPr-ZXX99369, NQWB01000005
Providencia alcalifaciens RIMD 1656011, JALB01000004
Providencia alcalifaciens R90-1475, JALC01000021
Providencia alcalifaciens F90-2004, JACS01000001
Providencia alcalifaciens 205/92, JALD01000003
Providencia alcalifaciens PAL-2, JAKZ01000003
Providencia alcalifaciens FDAARGOS 408, CP023536
Providencia alcalifaciens TYPE DSM 30120, EU587048
Providencia alcalifaciens Dmel2, AKKM01000030
Providencia alcalifaciens PAL-3, AZYZ01000005
Providencia alcalifaciens PAL-1, JALA01000025
Providencia stuartii INSRA21868, LGYB01000007
Providencia stuartii FDAARGOS 87, JTAU01000001
Providencia stuartii PS71, MSAA01000006
Providencia stuartii 50655837, LNHS01000070
Providencia stuartii YD789-2, PGGX01000012
Providencia stuartii AR 0026, CP026704
Providencia stuartii MRSN 2154, CP003488
Providencia stuartii FDAARGOS 145, CP014024
Providencia stuartii ATCC 33672, CP008920
Providencia stuartii BE2467, CP017054
Providencia stuartii TYPE DSM 4539, EU587025
Providencia stuartii ATCC 25827, ABJD02000046
Providencia stuartii FDAARGOS 294, NJEY02000002
Providencia stuartii Crippen, LVIE01000201
Providencia thailandensis TYPE C1112, KC447299
Providencia sneebia TYPE DSM 19967, EU587096
Providencia sneebia A75, EU587054
Providencia sneebia A91, EU587060
Providencia sneebia A102, EU587072
Providencia sneebia A101, EU587066
Providencia burhodogranariea TYPE DSM 19968, EU587102
Providencia burhodogranariea B97, EU587084
Providencia burhodogranariea B18, EU587078
Providencia burhodogranariea D, EU587114
Providencia burhodogranariea D37, EU587090
Providencia rustigianii TYPE DSM 4541, EU587031
Providencia heimbachae P12672, FIZD01000005
Providencia heimbachae TYPE DSM 3591, EU587019
Proteus mirabilis ATCC 29906, ACLE01000011

S3 Fig. *fusA* gene NJ tree. Neighbor Joining (NJ) phylogeny of *Providencia* bacteria as reconstructed from *fusA* gene sequences. Terminal branches are labelled by genus, species and strain designations as well as GenBank accession numbers. Numbers on branches indicate bootstrap support values. The size bar corresponds to 1 % sequence divergence. An orthologous sequence from the closely related bacterium *Proteus mirabilis* has been used as outgroup.
