# Supplementary material for: Providencia entomophila sp. nov., a new bacterial species associated with major olive pests in Tunisia
Source: PLoS One. 2019 Oct 22;14(10):e0223943. doi: 10.1371/journal.pone.0223943 (PMC6805009; doi:10.1371/journal.pone.0223943)
Supplement: S4 Fig — Neighbor Joining (NJ) phylogeny of Providencia bacteria as reconstructed from gyrB gene sequences. Terminal branches are labelled by genus, species and strain designations as well as GenBank accession numbers. Numbers on branches indicate bootstrap support values. The size bar corresponds to 1% sequence divergence. An orthologous sequence from the closely related bacterium Proteus mirabilis has been used as outgroup. (PDF) [file pone.0223943.s005.pdf]

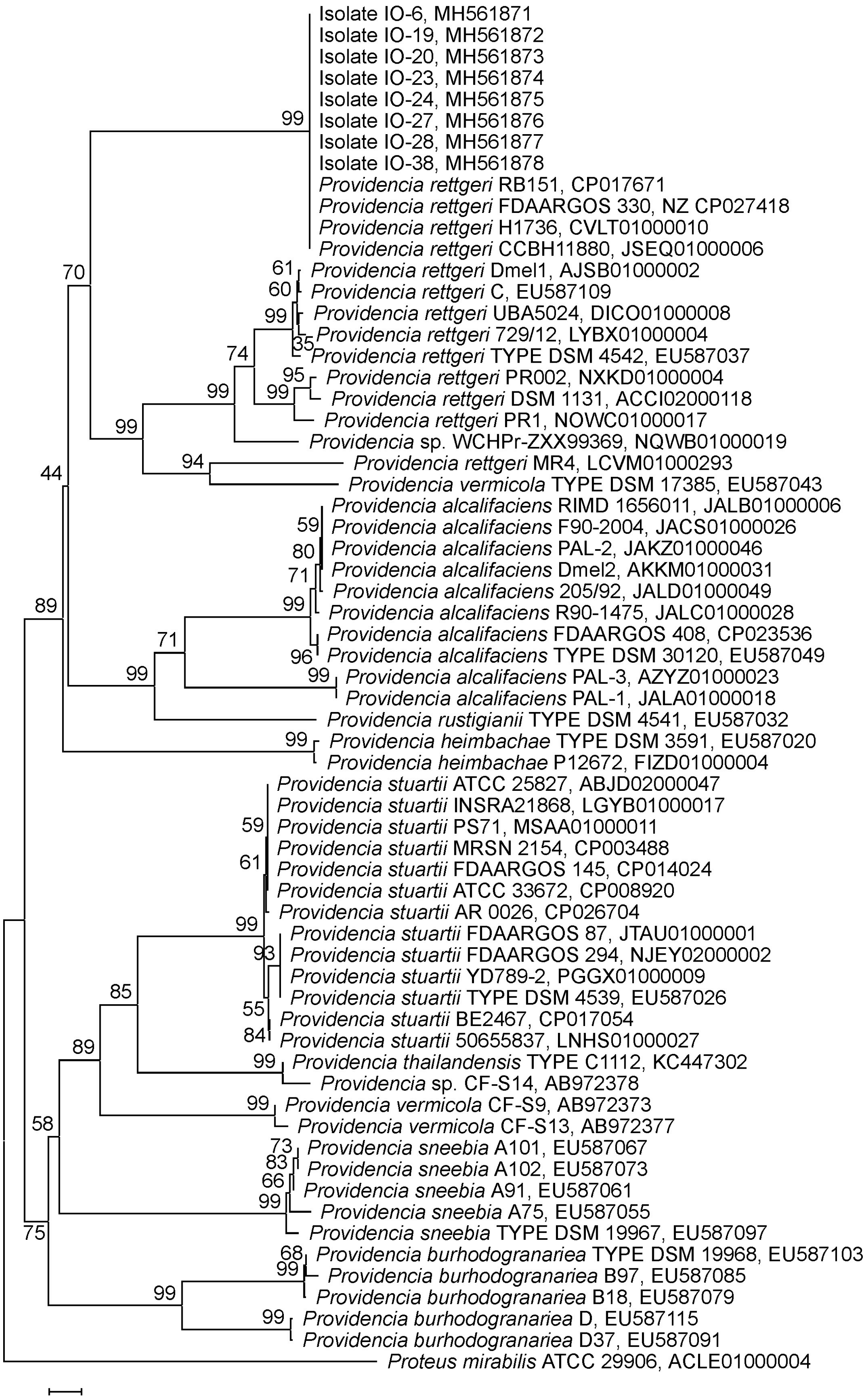
Isolate IO-6, MH561871
Isolate IO-19, MH561872
Isolate IO-20, MH561873
Isolate IO-23, MH561874
Isolate IO-24, MH561875
Isolate IO-27, MH561876
Isolate IO-28, MH561877
Isolate IO-38, MH561878
Providencia rettgeri RB151, CP017671
Providencia rettgeri FDAARGOS 330, NZ CP027418
Providencia rettgeri H1736, CVLT01000010
Providencia rettgeri CCBH11880, JSEQ01000006
Providencia rettgeri Dmel1, AJSB01000002
Providencia rettgeri C, EU587109
Providencia rettgeri UBA5024, DICO01000008
Providencia rettgeri 729/12, LYBX01000004
Providencia rettgeri TYPE DSM 4542, EU587037
Providencia rettgeri PR002, NXKD01000004
Providencia rettgeri DSM 1131, ACCI02000118
Providencia rettgeri PR1, NOWC01000017
Providencia sp. WCHPr-ZXX99369, NQWB01000019
Providencia rettgeri MR4, LCVM01000293
Providencia vermicola TYPE DSM 17385, EU587043
Providencia alcalifaciens RIMD 1656011, JALB01000006
Providencia alcalifaciens F90-2004, JACS01000026
Providencia alcalifaciens PAL-2, JAKZ01000046
Providencia alcalifaciens Dmel2, AKKM01000031
Providencia alcalifaciens 205/92, JALD01000049
Providencia alcalifaciens R90-1475, JALC01000028
Providencia alcalifaciens FDAARGOS 408, CP023536
Providencia alcalifaciens TYPE DSM 30120, EU587049
Providencia alcalifaciens PAL-3, AZYZ01000023
Providencia alcalifaciens PAL-1, JALA01000018
Providencia rustigianii TYPE DSM 4541, EU587032
Providencia heimbachae TYPE DSM 3591, EU587020
Providencia heimbachae P12672, FIZD01000004
Providencia stuartii ATCC 25827, ABJD02000047
Providencia stuartii INSRA21868, LGYB01000017
Providencia stuartii PS71, MSAA01000011
Providencia stuartii MRSN 2154, CP003488
Providencia stuartii FDAARGOS 145, CP014024
Providencia stuartii ATCC 33672, CP008920
Providencia stuartii AR 0026, CP026704
Providencia stuartii FDAARGOS 87, JTAU01000001
Providencia stuartii FDAARGOS 294, NJEY02000002
Providencia stuartii YD789-2, PGGX01000009
Providencia stuartii TYPE DSM 4539, EU587026
Providencia stuartii BE2467, CP017054
Providencia stuartii 50655837, LNHS01000027
Providencia thailandensis TYPE C1112, KC447302
Providencia sp. CF-S14, AB972378
Providencia vermicola CF-S9, AB972373
Providencia vermicola CF-S13, AB972377
Providencia sneebia A101, EU587067
Providencia sneebia A102, EU587073
Providencia sneebia A91, EU587061
Providencia sneebia A75, EU587055
Providencia sneebia TYPE DSM 19967, EU587097
Providencia burhodogranariea TYPE DSM 19968, EU587103
Providencia burhodogranariea B97, EU587085
Providencia burhodogranariea B18, EU587079
Providencia burhodogranariea D, EU587115
Providencia burhodogranariea D37, EU587091
Proteus mirabilis ATCC 29906, ACLE01000004
99
70
61
60
99
35
74
95
99
99
99
94
44
59
80
71
99
71
96
99
99
89
99
59
61
99
93
55
84
85
89
99
99
58
73
83
66
99
75
68
99
99
99

S4 Fig. *gyrB* gene NJ tree. Neighbor Joining (NJ) phylogeny of *Providencia* bacteria as reconstructed from *gyrB* gene sequences. Terminal branches are labelled by genus, species and strain designations as well as GenBank accession numbers. Numbers on branches indicate bootstrap support values. The size bar corresponds to 1 % sequence divergence. An orthologous sequence from the closely related bacterium *Proteus mirabilis* has been used as outgroup.
