# Supplementary material for: Providencia entomophila sp. nov., a new bacterial species associated with major olive pests in Tunisia
Source: PLoS One. 2019 Oct 22;14(10):e0223943. doi: 10.1371/journal.pone.0223943 (PMC6805009; doi:10.1371/journal.pone.0223943)
Supplement: S7 Fig — Neighbor Joining (NJ) phylogeny of Providencia bacteria as reconstructed from leuS gene sequences. Terminal branches are labelled by genus, species and strain designations as well as GenBank accession numbers. Numbers on branches indicate bootstrap support values. The size bar corresponds to 1% sequence divergence. An orthologous sequence from the closely related bacterium Proteus mirabilis has been used as outgroup. (PDF) [file pone.0223943.s008.pdf]

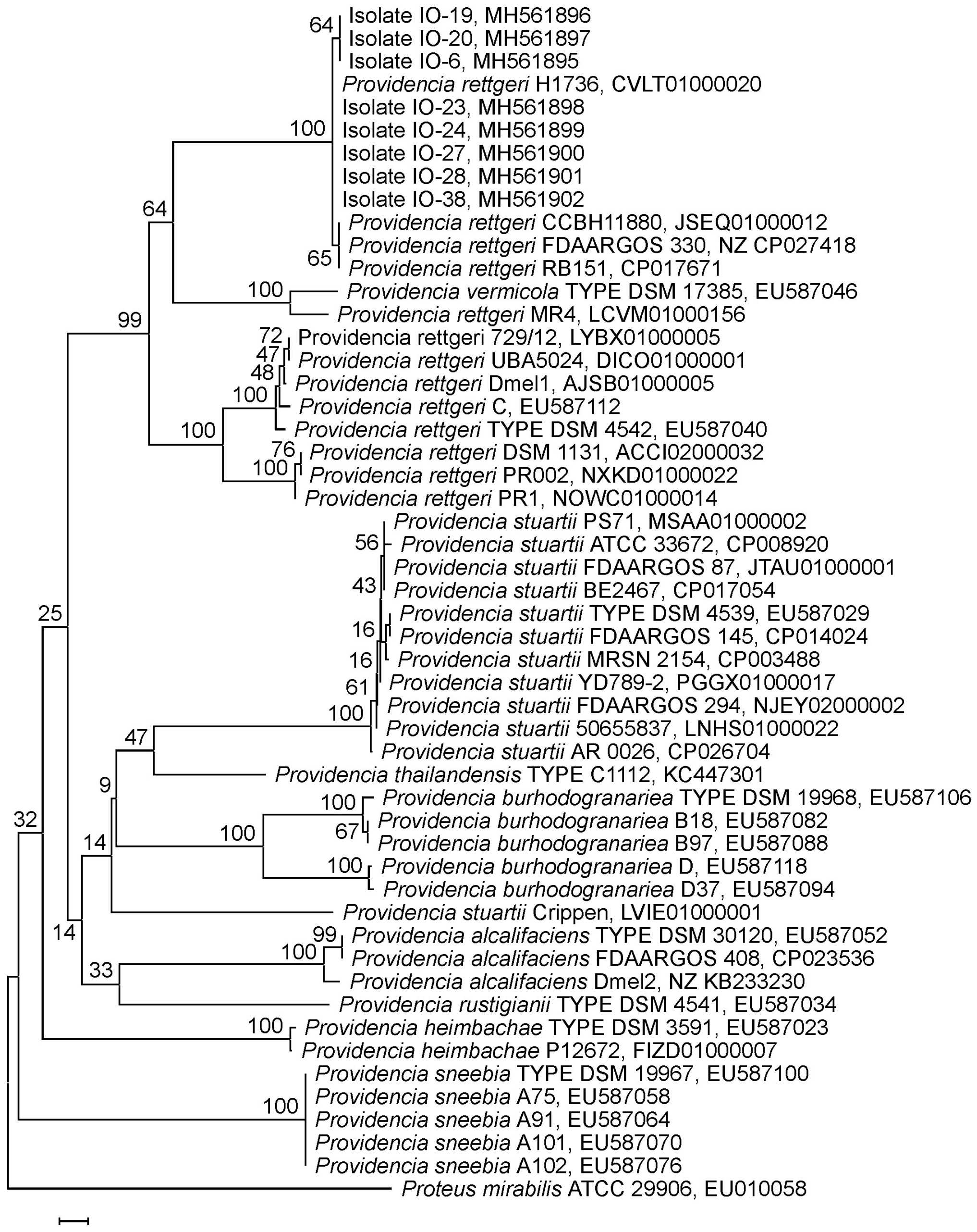

S7 Fig. *leuS* gene NJ tree. Neighbor Joining (NJ) phylogeny of *Providencia* bacteria as reconstructed from *leuS* gene sequences. Terminal branches are labelled by genus, species and strain designations as well as GenBank accession numbers. Numbers on branches indicate bootstrap support values. The size bar corresponds to 1 % sequence divergence. An orthologous sequence from the closely related bacterium *Proteus mirabilis* has been used as outgroup.
