# Supplementary figures and images for: Providencia entomophila sp. nov., a new bacterial species associated with major olive pests in Tunisia
Source: PLoS One. 2019 Oct 22;14(10):e0223943. doi: 10.1371/journal.pone.0223943 (PMC6805009; doi:10.1371/journal.pone.0223943)

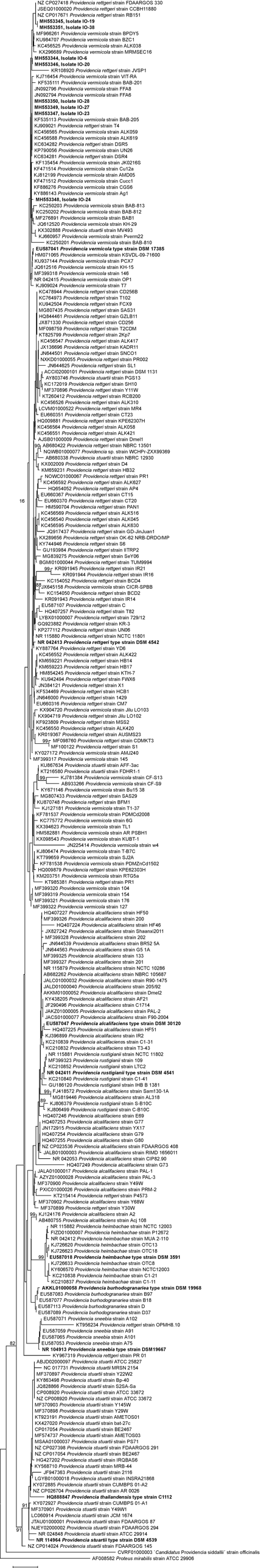

Supplement: S8 Fig — Neighbor Joining (NJ) phylogeny of Providencia bacteria as reconstructed from 16S ribosomal RNA encoding sequences. Terminal branches are labelled by GenBank accession numbers as well as genus, species and strain designations. Numbers on branches indicate bootstrap support values >80%. The size bar corresponds to 1% sequence divergence. An orthologous sequence from the closely related bacterium Proteus mirabilis has been used as outgroup. (TIF) [file pone.0223943.s009.tif]
